# Drivers and Obstacles of Consumers’ Continuous Participation Intention in Online Pre-Sales: Social Exchange Theory Perspective

**DOI:** 10.3390/bs14111094

**Published:** 2024-11-14

**Authors:** Ya Wang, Xiaodong Qiu, Jiwang Yin, Liya Wang, Rong Cong

**Affiliations:** 1 School of Economics and Management, Beijing Jiaotong University, Beijing 100044, China; xdqiu@bjtu.edu.cn (X.Q.); 19113075@bjtu.edu.cn (J.Y.); 21113077@bjtu.edu.cn (L.W.); 21113074@bjtu.edu.cn (R.C.); 2 Research Center for Central and Eastern Europe, Beijing Jiaotong University, Beijing 100044, China

**Keywords:** online pre-sale, perceived benefits, perceived costs, product type, continuous participation intention

## Abstract

This study focuses on the factors influencing consumers’ continuous participation willingness in online pre-sale activities. Based on perceived value and social exchange theory, it analyzes how perceived benefits (including practical benefits, hedonic benefits, and social benefits) and perceived costs (including search costs, waiting costs, and adjustment costs) affect consumer satisfaction and their continuous participation intention in online pre-sales. A total of 527 valid questionnaires were collected, and structural equation modeling was used for data analysis. The results indicate that perceived benefits are significantly positively correlated with consumer satisfaction and their willingness to continue participating, while perceived costs are significantly negatively correlated with both aspects. Additionally, the study found that product type moderates the effect of perceived benefits and perceived costs on consumer satisfaction. This research helps retailers understand consumers’ willingness to continuously participate in online pre-sales and provides valuable insights for developing pre-sale strategies.

## 1. Introduction

Online pre-sale is a marketing model in which, before the start of the official selling of products or services, merchants offer consumers the opportunity to purchase them online in advance [1]. Generally, merchants usually offer some promotional discounts in online pre-sale activities to stimulate consumers’ continuous participation, such as product discounts and gift provisions [2,3]. This model enables both consumers and merchants to achieve a “mutually beneficial” outcome [4]. Consumers can not only enjoy the promotional benefits of online pre-sales and the psychological satisfaction derived from early booking but also avoid the risk of stockouts during peak sales seasons [5]. On the other hand, suppliers can know consumer preferences based on the pre-sale performance of various products, enabling more rational arrangements for subsequent production and operations [6]. With the popularity of online promotion festivals such as Double Eleven and 618, online pre-sale activities have become one of the essential events for e-commerce to pre-heat the market and boost sales [7].

However, existing academic research on online pre-sales predominantly adopts the perspective of merchant operation management, investigating aspects such as the pricing strategy for pre-sale products [8], methods for maximizing inventory reduction and optimizing replenishment decisions [7], predicting future consumer demand [9], etc. These studies usually treat the impacts of online pre-sales on consumer demand and behavior as established facts [10,11], without considering the willingness and behavior of consumers in participating in online pre-sale activities.

At present, many e-commerce enterprises utilize social media or Web 2.0 as marketing tools [12]. They disseminate pre-sale information, promotional strategies, and other content through social media platforms such as Weibo, WeChat, and TikTok [13]. This approach can increase the exposure of pre-sale activities and attract the attention of numerous potential consumers [14]. Simultaneously, consumers can share and acquire valuable information through various social interaction methods such as user reviews, order sharing, and live streaming interactions. The gathered information can support their decision-making process and purchasing behavior [15]. While some scholars have considered consumers’ regret behavior [8] and return behavior [16] when studying retailers’ design of pre-sale programs, there is a lack of empirical research on the factors influencing consumers’ continuous participation intention from the consumer psychology perspective on online pre-sales. Considering the fact that online pre-sales present consumers with unique challenges, such as advance purchase commitments, heightened uncertainty due to early purchasing, limited product availability, and the exclusivity of pre-sale offers. These exacerbate the importance of perceived benefits (e.g., gaining early access to a limited number of items or discounts) and perceived costs (e.g., the risk of dissatisfaction or product delays), leading to a different pattern of motivation for sustained consumer engagement intentions in online pre-sales than that observed in standard online sales. Therefore, it is necessary to extend current traditional models of consumer behavior by examining how these unique conditions influence continuous participation intentions.

In fact, consumers’ continuous participation in online pre-sale activities can be regarded as an exchange behavior [17]. When consumers perceive benefits from online pre-sale activities, they will reciprocate by maintaining their participation. Therefore, based on social exchange theory, this study divides consumers’ perceived value in the online pre-sale process into perceived costs and perceived benefits [18]. By combining the various benefits that consumers derive from online pre-sale activities (such as price discounts, enjoyable shopping experiences, and a sense of identity) and the potential negative impacts of consumer product valuation uncertainty and extended pre-sale periods, we categorize perceived benefits into utility, hedonic and social benefits, and perceived costs into search, waiting and adjustment costs. We then further investigate their impact on consumers’ continuous participation in online pre-sale activities. Perceived benefits and perceived cost constitute the core components of the consumer decision-making process [19,20]. This study not only enriches the empirical research on the factors influencing consumer continuous participation intention in online pre-sales but also assists e-commerce enterprises in better understanding the psychological activities underpinning consumers’ continuous participation in online pre-sale activities, thereby enhancing the influence and attractiveness of online pre-sales.

The structure of this study is organized as follows: The literature review section discusses prior academic research on online pre-sales, explains the relationship between consumers’ perceived value and their continuous willingness to participate, and introduces social exchange theory as a theoretical foundation. In Section 3, we propose the theoretical model and research hypotheses. Section 4 presents the research methods, followed by a discussion of the research results and conclusions. The final section summarizes the contributions, limitations and future research directions of this study.

## 2. Theoretical Framework and Literature Review

### 2.1. Online Pre-Sales

Research on pre-sales first appeared in the airline industry, hotel industry, and specialized areas such as real estate [21]. With the continuous development of information technology, the pre-sale model has become increasingly common in the retail industry [7,22]. Due to the popularity of online shopping and consumers’ demand for diversified consumption processes, online promotional strategies have become increasingly diverse. Among them, online pre-sales have aroused considerable interest from major e-commerce companies [23].

Generally, the pre-sale model allows consumers to acquire products at a discount, which is not offered during the regular sales phase [6]. Furthermore, it enables them to establish product ownership earlier and avoid potential stock-out issues during the normal sales phase [5]. Nevertheless, consumers typically need to commit to a purchase and endure a waiting period before receiving the goods. For retailers, pre-ordering products by consumers can reduce inventory risk [7] and increase cash liquidity [24]. Moreover, consumers’ pre-orders in the online pre-sale can help retailers to predict consumer demand during the regular sales phase [6], thereby facilitating customized demand, on-demand production, and scale production. In addition, pre-sales separate the consumers’ purchasing phase from the product or service experience phase, introducing various uncertainties, including uncertainty in the consumers’ valuation of the product and uncertainty of their purchase necessity [25]. These factors may contribute to an increase in consumer purchases, consequently generating more orders for the retailer [1,16].

Existing research on online pre-sales primarily concentrates on the domain of business operations management. For instance, [9] verified that pre-sale orders can predict consumer demand, albeit assuming that the influence of online pre-sales on consumer demand is an established fact [10]. Nevertheless, in an online pre-sale marketing environment, customers often exhibit complex psychological perceptions, and not all consumers are eager to participate in the pre-sale process [26]. Although some researchers have explored the use of trade-in strategies to attract new and existing customers to engage in pre-sales [27], the changes in psychological perceptions during consumers’ participation in pre-sales remain unexplored.

Therefore, to fill in the gap of empirical research on consumers’ willingness to continue participating in online pre-sale scenarios, this study, based on social exchange theory, establishes a framework for analyzing perceived value to investigate the perceived costs and benefits associated with consumers’ involvement in online pre-sale activities.

### 2.2. Perceived Value and Continuous Participation Intention

Consumers’ continuous participation intention serves as a crucial indicator for evaluating the attractiveness of online pre-sale activities. [28] was the first to introduce the concept of continuous intention to participate, defining it as a consumer’s tendency to reuse a particular system. This intention shows that despite the efforts to promote their products over competing businesses, customers consistently exhibit a preference for revisiting a particular company or persistently utilizing its services [29]. In the context of online pre-sales, we define consumers’ continuous participation intention as the likelihood of their ongoing involvement in such activities. This includes behaviors such as repeatedly taking advantage of early access discounts or pre-ordering products. When consumers demonstrate a dependency on and willingness to recommend products and services for online pre-sales, we believe they have the intent to continue to participate in these activities [30]. Consumers’ continuous participation intention is influenced by various factors, such as perceived value, satisfaction with consumption experience, and so on [31,32].

Previous studies have demonstrated that perceived value plays a vital role in developing long-term relationships with consumers [33] and serves as an effective way to measure consumer satisfaction and continuous intention [34]. Perceived value, which is a concept based on consumers’ subjective perceptions, reflects their attitudes and assessments of products or services [35]. Previous studies have validated that perceived value has a direct and significant impact on consumer behavior in various scenarios [33,36]. For online pre-sale activities, consumers’ continuous participation also plays a key role in achieving the expected goals [37]. In a situation where diversified promotion strategies are adopted to compete for existing consumers, customer retention becomes even more critical. However, there is a lack of research on the relationship between perceived value and consumers’ continuous participation willingness within the emerging online pre-sale sales model. Therefore, it is of great significance to explore the impact of perceived value on consumers’ continuous participation in the online pre-sale.

An online pre-sale, which is a promotional strategy originating from the Internet, can bring consumers convenience and utility-based benefits, allowing them to obtain products in advance and enjoy exclusive discounts [38]. Additionally, it provides shopping pleasure and more different experiences [39], allowing consumers to enjoy the pleasure of early purchases and the excitement of receiving new products. Moreover, engaging in online pre-sale activities can provide consumers with first-hand product experience and enhance their interactions with others by sharing product information. Consequently, online pre-sale activities help consumers realize their self-worth and obtain the social benefits of constructing a social identity [40]. The perceived benefits of consumer participation in pre-sale activities are accompanied by certain costs. Perceived costs do not solely include transactional expenses but also encompass hidden costs, such as time and effort [41]. Due to the potential scarcity of information during the pre-sale process and the difficulty in discerning product quality among numerous sellers [23], consumers may spend more time and effort gathering information about pre-sale products and comparing different products and services, especially for limited edition or customized items. Consumers may also have to bear the costs associated with the extended waiting period. Furthermore, the uncertainties regarding future demand and product valuations within the online pre-sale [22] may result in difficulties or inconvenience.

In a pre-sale context, this paper attempts to categorize the perceived benefits into three dimensions: utility, hedonic, and social benefits [42,43]. Likewise, perceived costs are classified into three dimensions: search, waiting, and adjustment costs [44,45,46]. This approach is adopted to investigate the impact of each dimension of perceived benefits and perceived costs in the online pre-sale on consumers’ continuous participation intention.

### 2.3. Social Exchange Theory

Social exchange theory has been extensively used to demonstrate individual behavior in various domains, such as consumer behaviors [47,48,49]. Social exchange theory holds that an individual’s behavior can be understood as a social exchange [40], and a consumer’s engagement in transactional behavior is contingent upon the comparison of benefits gained and costs incurred in the transaction [50]. In social exchange theory, exchange can include tangible benefits (e.g., money) and intangible benefits (e.g., social services and interpersonal interaction) [40]. Ref. [51] emphasizes that intangible factors in social exchange may be more important than tangible resources, as they impact the structure of relationships between groups more directly. In the context of e-commerce, both parties anticipate receiving feedback within the exchange relationship, thereby enabling the continuation of the exchange cycle. Over time, and with the occurrence of more exchanges, this exchange cycle is reinforced [19]. Guided by the principle of reciprocity, when an individual perceives that the behavior performed is valuable, they will be more inclined to repeat it, i.e., the consumer is motivated to purchase, recommend, and continue to participate in reciprocal reward behaviors [52]. Specifically, within the online pre-sale mode, consumers’ continuous participation can also be regarded as the reciprocity of the online pre-sale activities [17]. As consumers perceive economic and social benefits from participating in online pre-sale activities, their engagement in such activities will increase.

Therefore, we adopt social exchange theory as the theoretical foundation, categorize perceived value into perceived costs and perceived benefits, and establish an analytical framework for perceived value. Utilizing this framework, we examine the factors influencing consumers’ willingness to maintain participation in pre-sale activities during an online pre-sale. Furthermore, we have taken into account the moderating effect of product type within our research framework, given that differences in product types can affect consumer decision-making processes during online pre-sales.

## 3. Research Hypothesis Development

**Figure 1:**
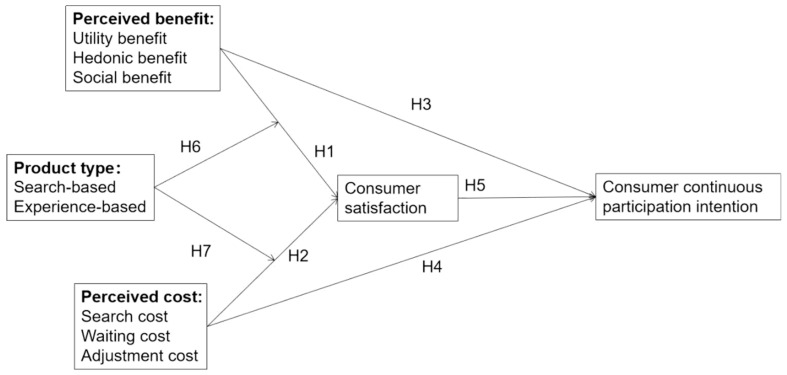
Study model diagram.

Based on the above discussion, we adopted social exchange theory and developed a research model (Figure 1) in which consumers’ continuous participation intention in the online pre-sale from the perspective of perceived benefits and perceived costs is explored. In addition, we considered the moderating effect of the product type.

### 3.1. Perceived Benefits/Cost and Consumer Satisfaction

Consumer satisfaction refers to a consumer’s delighted response and serves as a measure of the extent to which consumers find products or services meet their needs [42]. Many studies have shown that perceived benefits have a positive impact on consumer satisfaction [34,53]. In this study, we consider three perceived benefits associated with an online pre-sale: utility benefit, hedonic benefit, and social benefit.

Utility benefits refer to the functional and practical benefits that consumers obtain by participating in online pre-sale activities [54]. These benefits include providing consumers with cost savings, convenience, a variety of choices during the purchase of a product or service, and so on [55]. In pre-sale activities, online retailers often use price discounts to attract consumers’ attention [56], and the low prices do help consumers to have a positive attitude towards participating in online shopping [57]. Therefore, participation in online pre-sale activities helps consumers find deals and discounts [58], which may enhance their satisfaction with participating in the online pre-sale activities.

Hedonic benefits focus on the emotional benefits to consumers, referring to the non-utility benefits that consumers obtain in the process of purchasing a product or service [59]. Hedonic benefits are often related to the level of interest that consumers experience during shopping, such as the excitement and enjoyment that consumers generate and the pleasurable experience they obtain [60]. Online pre-sales allow consumers to try out new products or services and unique experiences [7]. In the process of online pre-sale activities, consumers can enjoy the pleasure of buying in advance, explore the fun of shopping, and meet their internal needs [61], hence generating positive emotions and improving satisfaction [59].

Social benefits refer to the utility of social self-concept obtained during the purchase of a product or service [29], such as providing others with first-hand product experience, enhancing social interaction with others and the sense of belonging to social groups [40,62]. Consumers may also share their experiences of participating in pre-sale activities on social media, and recommend good products to others. This social interaction can resonate with other consumers [29,63], thus enhancing consumers’ sense of status and identity and making consumers feel social benefits [59]. When consumers truly feel the social approval or a higher affective state from such social interaction experiences, their satisfaction will be further enhanced [64].

When consumers participate in online pre-sale activities, the perceived benefits, as a positive psychological factor, will prompt individuals to have a good feeling about their participation experience [65]. Based on this, we propose the following hypotheses:

**H1a.**  *Perceived utility benefit is positively correlated with consumer satisfaction.*

**H1b.**  *Perceived hedonic benefit is positively correlated with consumer satisfaction.*

**H1c.**  *Perceived social benefit is positively correlated with consumer satisfaction.*

When consumers participate in online pre-sale activities, there’s a possibility of experiencing lower-than-expected pleasure. Moreover, costs associated with consumption can directly affect satisfaction [53]. Perceived cost, which refers to the price paid by users to obtain benefits, serves as a trade-off concept of perceived benefit. The higher the perceived cost to the user, the lower the user’s satisfaction, and consequently, the lower the willingness to continue using the service [45,66]. This study considers three perceived costs associated with an online pre-sale: search cost, waiting cost, and adjustment cost.

Search cost refers to the time and energy required by consumers in searching for information about price, quality, and other attributes to compare when they want to purchase a product or service [67]. In an information overload environment, it tends to lead to the problem of choice complexity. Also, consumers need to spend more time and make more effort comparing different goods, or go to physical stores to experience the product to reduce their uncertainty about the product or service [7], which leads to more informed decisions. However, due to the limited information processing ability of consumers, they feel cognitive burden, which leads to negative emotions and affects their satisfaction [68].

The losses caused as a result of consumers’ waiting are called waiting costs. Studies have shown that long waiting times significantly reduce consumer satisfaction [69]. Consumers may feel anxious, dissatisfied, and disappointed during the waiting process. Therefore, these negative emotions affect their overall evaluation of the product or service [70]. When consumers suffer from a waiting loss, the utility of their purchase decreases. [71] argued that customer satisfaction decreases as customer perception of waiting time increases.

Adjustment costs refer to the time and energy required by consumers on order changes and post-sale communication with merchants that occur during the purchase process [45]. Consumers always face uncertain product valuation (such as product quality, function, etc.) and delivery time during the pre-sale period [7]. Therefore, they may need to modify their pre-sale orders or consult the merchant for various reasons, which will involve a series of tedious processes. This may require consumers to invest more time and effort, thus increasing adjustment costs. This additional investment often leads to consumer dissatisfaction and frustration [72].

Based on the above, we propose the following hypotheses:

**H2a.**  *Perceived search cost is negatively correlated with consumer satisfaction.*

**H2b.**  *Perceived waiting cost is negatively correlated with consumer satisfaction.*

**H2c.**  *Perceived adjustment cost is negatively correlated with consumer satisfaction.*

### 3.2. Perceived Benefits/Cost and Continuous Participation Intention

The motivation for consumers’ continuous participation intention in online pre-sale activities may be their perceived benefits (i.e., utility benefits, hedonic benefits, and social benefits) [58], which reflect their subjective perceptions of the product or service. In online pre-sale activities, consumers often enjoy lower prices than regular prices and more personalized choices [73]. Once consumers acquire valuable products and services, they are likely to have a positive view of pre-sale activities [74] and be more willing to continue participating to obtain more valuable products and services [29]. Hedonic benefits focus on consumers’ experience level when participating in online pre-sale activities. The hedonistic atmosphere of the shopping environment can highly stimulate consumers’ positive emotions [75], thus making consumers feel entertained [76], which motivates them to continuously participate in activities [77]. Ref. [38] also found that hedonic benefits have a significantly positive impact on consumers’ repurchase intentions through empirical research. Previous research has shown that social benefit is an important predictor of behavioral intentions: the higher the perceived social benefits, the stronger behavioral intentions [61,78]. The authors of Ref. [79] believe that, in the process of participation, reputation or social prestige can be achieved, and the satisfaction from helping others and feeling valued can also be derived. These gratifications can positively influence consumer attitudes, thus likely leading to more frequent and extended periods of participation. In online pre-sale activities, when consumers share common topics with other consumers or share their values, they resonate with each other, which enhances consumers’ sense of identity and belonging. This makes them more likely to continue participating in online pre-sale activities [29].

Therefore, we propose the following hypotheses:

**H3a.**  *Perceived utility benefit is positively correlated with consumer continuous participation intention.*

**H3b.**  *Perceived hedonic benefit is positively correlated with consumer continuous participation intention.*

**H3c.**  *Perceived social benefit is positively correlated with consumer continuous participation intention.*

Perceived cost is a key factor influencing consumers’ continuous willingness. [41] argue that reducing consumers’ information search costs can enhance their repurchase intentions. In online pre-sale activities, consumers need to invest considerable time and effort searching for and comparing product information. Higher search costs may cause consumers to feel confused and uneasy [68], triggering anticipated regret behavior and thereby affecting their willingness to continue participating in online pre-sale activities [80]. Pre-sale activities usually require consumers to pay in advance and receive products or services at a future point in time [5]. The uncertainty in the waiting process may also increase consumers’ anxiety [71]. Higher waiting costs might prompt consumers to seek alternative options to meet immediate needs [81], thereby affecting their continuous participation intention. In addition, if consumers believe that the adjustment cost of participating in pre-sale activities is too high, they may perceive pre-sale activities as unattractive and choose to discontinue their participation [41].

Based on the above, we propose the following hypotheses:

**H4a.**  *Perceived search cost is negatively related to consumer continuous participation intention.*

**H4b.**  *Perceived waiting cost is negatively related to consumer continuous participation intention.*

**H4c.**  *Perceived adjustment cost is negatively related to consumer continuous participation intention.*

### 3.3. Consumer Satisfaction and Continuous Participation Intention

Continuous participation intentions are influenced by consumer satisfaction [32]. Extensive prior research has demonstrated that consumer satisfaction affects consumer behavior: the more satisfied users are, the more willing they are to repurchase products or continue using services [82,83]. In a study on mHealth services, [84] highlighted the positive effect of satisfaction on continued intention. [85] confirmed a significant positive relationship between user satisfaction and continued intention to use online customer service in e-commerce. In the context of relationship marketing and customer retention, customer satisfaction has consistently been emphasized, as it influences consumer motivation and subsequent behavioral intentions [49]. In an online pre-sale, we assume that consumer satisfaction is closely related to consumers’ continuous participation intentions. Based on social exchange theory, if consumers feel satisfied with pre-sale activities, they may choose to continue engaging in online pre-sale activities at later stages. This could generate reciprocal rewarding behaviors such as purchasing, recommending, and maintaining participation.

Based on the above discussion, we propose the following hypothesis:

**H5.**  *Consumer satisfaction has a positive effect on consumer continuous participation intention in an online pre-sale.*

### 3.4. Moderating Effect of Product Type

Ref. [86] classified products into two categories: search-based products and experience-based products. The primary distinction between search-based and experience-based products lies in whether consumers can evaluate the product attributes before making a purchase [87]. In order to better understand consumer decision-making in online pre-sales, we draw on research from [86] to characterize product types as search based or experience based.

Search-based products have relatively objective attributes (e.g., storage capacity, size, and performance) that are easy to compare and evaluate. They can be quantified without personal experience [88]. For this type of product, consumers can gather information through various channels before making a purchase. Once enough information is collected, they can understand the product’s performance and attributes more accurately and objectively, forming a relatively homogeneous opinion of the product. Examples of search-based products include mobile phones, home appliances, books, etc. In contrast, experience-based products have attributes (e.g., taste of food, touch of clothes) that are difficult to quantify [89], and there is no unified objective evaluation standard. As a result, product characteristics cannot be fully understood simply through information search alone. Instead, product quality and applicability must be evaluated through practical use and personal experience [88]. Examples of experience-based products include clothing, cosmetics, food products, etc.

The search/experience product classification paradigm has been widely applied in the study of consumer behavior in the context of the Internet [87,90,91]. This study examined whether and how product types moderate the effects of consumers’ perceived benefits and perceived costs on consumer satisfaction during an online pre-sale.

Price discounts in online pre-sale activities can often stimulate consumers’ desire to buy [92]. For search-based products, the comparison of quality and performance is more objective, with less price differentiation among similar types of products. In contrast, experience-based products are more personalized, with greater differences in consumers’ expected prices and perceived utility benefits, which makes the price mechanism more likely to play a key role. Consumers’ utility gains from purchasing different types of products can thus differ. Online pre-sale activities also provide a certain entertainment atmosphere, with the aim of attracting more consumers. Since experience-based products primarily tend to satisfy consumers’ emotional needs [93], in such an entertainment atmosphere, experience-based products are more likely to provide consumers with an enjoyable and pleasurable experience, and their hedonic benefits are also more obvious compared with search products. For pre-sale products, especially experience-based products, consumers are more inclined to refer to the feelings of other consumers, leading to more interaction with other users [94]. The social benefits derived from this interaction will also have a positive effect on consumer satisfaction.

Based on the above discussion, we propose the following hypotheses:

**H6a.**  *The product type has a moderating effect on the relationship between utility benefits and satisfaction. Compared to that in search-based products, the effect of utility benefits on satisfaction is more obvious in experience-based products.*

**H6b.**  *The product type has a moderating effect on the relationship between hedonic benefits and satisfaction. Compared to that in search-based products, the effect of hedonic benefits on satisfaction is more obvious in experience-based products.*

**H6c.**  *The product type has a moderating effect on the relationship between social benefits and satisfaction. Compared to that in search-based products, the effect of social benefits on satisfaction is more obvious in experience-based products.*

Due to the direct presentation of attribute information for search-based products, consumers require less time to obtain and process information. In contrast, the highly asymmetric information attributes of experience-based products make it necessary for consumers to search for information through multiple channels [95]. Especially for pre-sale products, consumers may need to combine information from different sources to determine the overall value of the product, thus reducing the uncertainty of purchasing the product [91,96]. Therefore, search costs have a more significant impact on experience-based products [97]. Ref. [98] showed that the search intensity of experiential products is at least three times higher than that of search products. Online pre-sales can reduce inventory costs for merchants, but there is also the possibility for merchants to purchase and produce after receiving orders from consumers. When consumers receive the product, the significance of the product may be reduced. Particularly for experience-based products, consider pre-sale dresses purchased in the summer as an example. If consumers do not receive the dresses until in the fall, which is after a long pre-sale period, the value and significance of the dresses will be reduced, for they cannot meet the current needs of consumers, thus inevitably affecting consumer satisfaction. Since experience-based products primarily involve personal experiences and feelings [99], consumers are more likely to adjust their orders due to personal preferences or unexpected events, which results in higher adjustment costs and lower consumer satisfaction.

Based on the above discussion, we propose the following hypotheses:

**H7a.**  *The product type has a moderating effect on the impact of search costs on consumer satisfaction. Compared to that in search-based products, the negative impact of search costs on consumer satisfaction is more obvious in experience-based products.*

**H7b.**  *The product type has a moderating effect on the impact of waiting costs on consumer satisfaction. Compared to that in search-based products, the negative impact of waiting costs on consumer satisfaction is more obvious in experience-based products.*

**H7c.**  *The product type has a moderating effect on the impact of adjustment costs on consumer satisfaction. Compared to that in search-based products, the negative impact of adjustment costs on consumer satisfaction is more obvious in experience-based products.*

## 4. Research Methods

### 4.1. Measurement Instrument

The measurement instrument for this study is a carefully designed questionnaire, mainly consisting of three parts. The first part briefly introduces the research background and objectives. Specifically, it briefly introduces online pre-sale activities to respondents so that those unfamiliar with this marketing method can have a general understanding, and those with experience in online pre-sale activities can reinforce their impressions. The second part collects the demographic information of the respondents, including their gender, age, occupation, etc. The third part aims to understand respondents’ views on the different research variables influencing their continuous participation intention in online pre-sale activities. Our research model includes eight research variables, whose measurement items are adapted from previously validated scales (items and sources are shown in Appendix A) and modified to ensure that each item is suitable for the current research context. All items are measured using a 7-point Likert scale, ranging from 1 to 7 (from “completely disagree” to “completely agree”). Following a previous study [100], based on the number of choices respondents made in the “product type” option, they were coded as 1 (experiential products) and 0 (search products).

To avoid response bias, before officially distributing the questionnaire, we sent it to some academic experts and invited them to review the questionnaire to ensure that the questions were clearly and accurately stated. We made corresponding modifications to the questionnaire based on their feedback to ensure that participants could accurately understand each question. Furthermore, to ensure the accuracy of the Chinese questionnaire and its consistency with the original English questionnaire, we adopted a back-translation method when designing the Chinese questionnaire.

### 4.2. Data Collection

This study collected data through an online survey platform (Wenjuanxing, www.wjx.cn). The survey was conducted in two phases, the first from October to November 2022 and the second from May to June 2023, which included the pre-sale stages of two important shopping festivals Double 11 and 618 in China’s e-commerce industry. Respondents were first invited to answer whether they had ever participated in an online pre-sale event, and those who answered yes were our target sample. A total of 639 questionnaires were collected. To ensure the validity of the questionnaires, we eliminated invalid questionnaires based on the following three criteria: (1) the completion time of the questionnaire was less than 1.5 min; (2) all answers had identical values; and (3) the specific question “must choose completely agree” in the questionnaire was answered incorrectly. After screening, 527 valid samples were obtained, with a sample recovery rate of 82.47%. Table 1 shows the demographic information of the samples. In the valid samples, males accounted for 41.7%, and the age of all respondents was mainly concentrated in the 19–25 age group (81.6%). Most of the subjects had received a good education, and nearly 80% of the subjects had more than 3 years of online shopping experience. The demographic characteristics in this article are also common in previous studies [101,102].

## 5. Data Analysis

This study collected experimental data through a questionnaire survey method. Compared to other methods, we chose Structural Equation Modeling (SEM) to model the relationships among latent variables, exploring the connections between perceived benefits, costs, consumer satisfaction, and continuous participation intention while accounting for measurement error in the structure. SEM is a method for building, estimating, and testing causal relationship models capable of handling complex relationships among multiple variables, including both observed and latent constructs. Additionally, SEM offers flexibility in simultaneously testing multiple hypotheses and assessing the model’s goodness of fit. SPSS 26 and AMOS 24 software were used for data analysis and empirical testing in this study.

### 5.1. Reliability and Validity Tests

In this study, the reliability and validity of the research data were assessed to ensure the dependability and accuracy of the subsequent research findings.

The results of the reliability measures depicted in Table 2 show that the Corrected Item Total Correlation (CITC) values for all of the indicators, except for consumers’ continuous participation intention, exceed 0.7, which meets the requirement of being greater than 0.3 [103]. Moreover, the Cronbach’s α values for most factors are above 0.9, with the consumers’ continuous participation intention having a Cronbach’s α value of 0.788, which is greater than 0.7. These results indicate that the reliability indicators for all variables meet the necessary requirements, demonstrating that the questionnaire possesses strong reliability [104].

**Figure 2:**
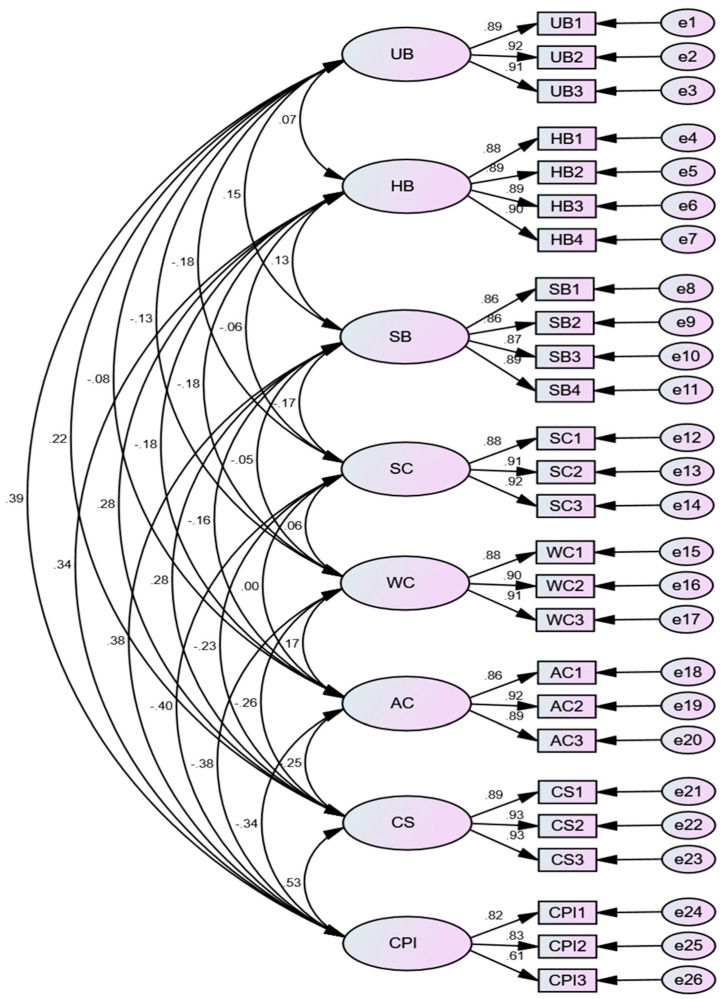
Confirmatory factor analysis CFA model diagram.

To verify the measurement model fitness, a confirmatory factor analysis was conducted on each variable dimension, as shown in Figure 2. The model fitness test results in Table 3 reveal that CMIN/DF = 1.193 < 3, RMSEA = 0.019 < 0.05, IFI = 0.995, TLI = 0.994, and CFI = 0.995, all of which reach excellent levels above 0.9. These findings indicate that the model in our study possesses good fitness [105]. The analysis results in Table 2 show that the Average Variance Extracted (AVE) values for each variable are above 0.5, and the Composite Reliability (CR) values are above 0.7. This demonstrates that each dimension exhibits good convergent validity and composite reliability [106]. Regarding the analysis of discriminant validity, the standardized correlation coefficients between any two variables are less than the square root of their corresponding AVE values (the main diagonal elements represent the square root of the AVE), indicating satisfactory discriminant validity between the variables.

### 5.2. Structural Model Path Analysis and Hypothesis Testing

**Figure 3:**
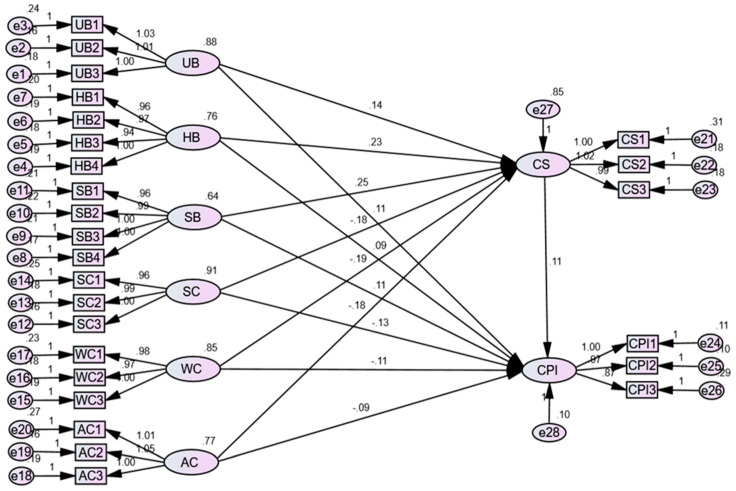
Structural equation model analysis diagram.

A structural equation model (Figure 3) was employed using AMOS 24 to investigate the impact of the three dimensions of perceived benefits and perceived costs on consumer satisfaction and consumers’ continuous participation intentions in an online pre-sale. The SEM results demonstrated a good fit for the model (CMIN = 425.779, DF = 286, CMIN/DF = 1.489, RMSEA = 0.030, IFI = 0.987, TLI = 0.985, and CFI = 0.987) [105]. Table 4 presents the path coefficients and their significance test results. According to Table 4, within the online pre-sale, the three dimensions of consumer perceived benefit have a significant positive effect on consumer satisfaction (β = 0.143, 0.227, and 0.249, respectively, *p* < 0.05) and consumers’ continuous participation intentions (β = 0.111, 0.085, and 0.109, respectively, *p* < 0.001). Conversely, the three dimensions of consumer perceived cost have a significant negative effect on both consumer satisfaction (β = −0.176, −0.195, and −0.18, respectively, *p* < 0.001) and consumers’ continuous participation intentions (β = −0.176, −0.195, and −0.18, respectively, *p* < 0.001). Additionally, satisfaction has a significant positive effect on consumers’ continuous participation intentions (β = 0.106, *p* < 0.001).

### 5.3. Moderating Effect Analysis and Hypothesis Testing

In this study, the research data were divided into two groups according to product types: the search-based product group (N = 289) and the experience-based product group (N = 238). Group regressions were performed on these two groups of datasets to obtain the path coefficients, standard errors and the significance of the path coefficients for the two models. A Z-test was employed to determine whether there were significant differences between the two groups of data with the test criteria defined as $Z=\frac{B_{1}-B_{2}}{\sqrt{SE_{1}^{2}+SE_{2}^{2}}}$ [107]. The multi-group analysis results in Table 5 indicate that product type has moderating effects on consumer utility benefit (|Z| = 2.338 > 1.96), hedonic benefit (|Z| = 3.363 > 3.29), and social benefit (|Z| = 3.664 > 3.29) on consumer satisfaction. Additionally, product type plays a moderating role in the impact of consumer search cost (|Z| = 3.105 > 2.58) on consumer satisfaction. For the effects of waiting cost (|Z| = 0.636 < 1.96) and adjustment cost (|Z| = 1.585 < 1.96) on consumer satisfaction, the moderating effect of product type is not significant, so hypotheses H7b and H7c are not supported.

**Figure 4:**
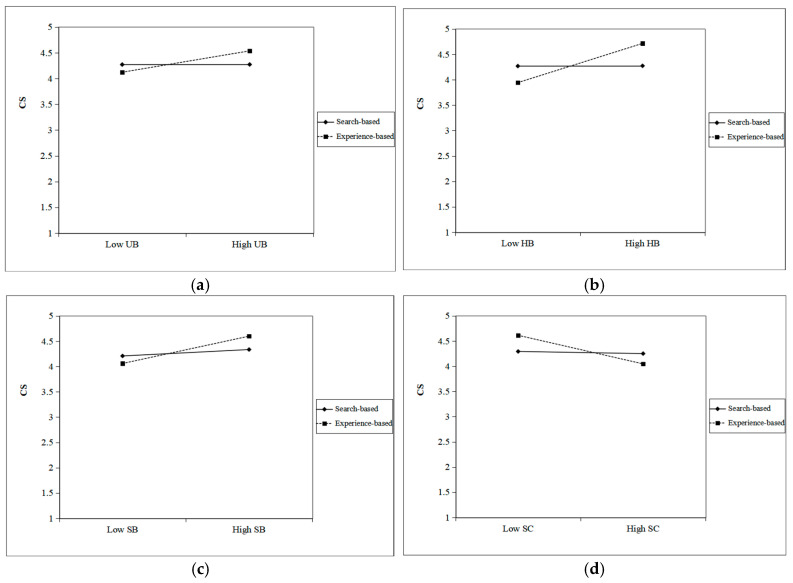
Moderating effect of product type on (**a**) UB, (**b**) HB, (**c**) SB, and (**d**) SC.

The moderating effect was further tested using hierarchical regression, yielding results consistent with the grouped regressions (Table 6). To further investigate the nature of the interaction effect, a simple slope analysis was performed, and the corresponding graphs are illustrated in Figure 4. Figure 4a–c reveal that for the three dimensions of perceived benefits, the positive effect is more obvious for experience-based products than for search-based products (i.e., a steeper slope for the relationship between the three dimensions of perceived benefits and consumer satisfaction), which indicates that hypotheses H6a-c are supported. Regarding the three dimensions of perceived costs, the moderating effect of product type is insignificant for the effects of waiting costs and adjustment costs on consumer satisfaction and significant only for the effects of search costs on consumer satisfaction. Figure 4d demonstrates that the negative effect of experience-based products on satisfaction is more obvious than that of search-based products, suggesting that hypothesis H7a is supported.

## 6. Discussion

In this study, we investigate the factors influencing consumers’ continuous participation intention in online pre-sales from a multidimensional perspective where perceived benefits and perceived costs in the social exchange theory are considered. In addition, we explore differences in the influences of perceived benefits and perceived costs on consumer satisfaction with various product types in online pre-sales.

The study found that utility benefits, hedonic benefits, and social benefits all have a significant positive effect on consumer satisfaction and consumers’ continuous participation intentions, which is consistent with previous research findings [79,102]. As hypothesized by H1a–c and H3a–c, when consumers receive tangible practical benefits, they will obtain higher perceived utility benefits. Likewise, users who perceive higher utility benefits tend to exhibit greater satisfaction and continuous participation intention. When consumers perceive enjoyment and a pleasant shopping experience during the shopping process, they are more likely to be satisfied with their shopping experience, and their continuous participation intention also increases. Additionally, when consumers share their shopping lists and experiences of pre-sale activities with others and consequently gain more attention and recognition, they can perceive an increase in social benefits. Such psychological satisfaction will positively affect consumer satisfaction, thus enhancing their intention to continuously participate in online pre-sale activities. In other words, when consumers perceive higher utility, hedonic, and social benefits in pre-sale activities, their satisfaction and continuous participation intention increase. This aligns with the “reciprocity principle” of social exchange theory, which suggests that when people perceive positive outcomes from an exchange, the exchange is more likely to be continued [108].

According to social exchange theory, individuals have a natural tendency to avoid harm [109]. As hypothesized in H2a–c and H4a–c, when consumers perceive that they are paying a greater cost, their satisfaction decreases, and their continuous participation intentions weaken. In the Internet era, the market environment is bombarded with information, providing consumers with diverse choices while increasing their information search costs [41]. In this scenario, customers experience mental and emotional tiredness and exhaustion, leading to emotional dissonance [110], which lowers consumer satisfaction and willingness to continue participating in activities [111]. Consumer waiting time is one of the determinants of satisfaction [23,112]. In the online pre-sale, there is a significant time lag between purchase and product delivery, with consumers often having to wait a long time to complete order delivery [23]. This may result in an unpleasant shopping experience for consumers [71], deterring them from participating in online pre-sale activities and purchasing pre-sale products again [113]. In online pre-sale activities, when the products and services received do not meet consumers’ expectations after an extended waiting period, consumers may choose to return or exchange the products or services [114]. The time and effort required in cancelling or changing orders can cause a decrease in consumer satisfaction, potentially discouraging future participation in online pre-sale activities.

Consumer satisfaction reflects a consumer’s psychological state throughout the consumption process after a product or service has been compared with the expectations of its value [115]. In an online pre-sale, consumers who feel satisfied with such a pre-sale shopping experience are more likely to continue participating in online pre-sales and recommend them to others. As predicted by H5, consumers’ continuous participation intention is stronger when their satisfaction is higher.

Furthermore, following [41], we evaluate the influence of product type. Our findings indicate that product type influences the relationship between consumers’ perceived benefits, perceived costs, and satisfaction. For experience-based products, online pre-sale promotions fulfill consumers’ desire for monetary savings, enjoyment, and value expression. The utility, hedonic, and social benefits that customers derive from participating in online pre-sale activities have a greater impact on satisfaction (H6a–c). This contrasts with the research of [95], who concluded that price discounts had a more significant influence on search-based products when customers shop online. Since search-based products have objective and easily comparable characteristics [91], consumers usually carry out a valuation of the product based on the objective attributes of search-based products during information gathering [98,116]. The prior sale records of the pre-sale products before large-scale sale promotion events or festivals are also available online [23], so when the price offered by pre-sale events is too low, it may increase consumers’ skepticism about product quality [57]. On the other hand, for experience-based products, the quality assessment process can be only conducted by searching for more consumer-shared experiences or by subsequent usage after purchasing [98], which results in higher search costs. This confirms H7a’s prediction that consumer search costs have a more negative influence on customer satisfaction for experience-based products.

The relationship between waiting and adjustment costs and customer satisfaction is not significantly influenced by the type of product (H7b and H7c do not hold). This non-significant moderating effect might result from the following reasons: Firstly, in online pre-sales, the separation of the purchase phase from the product or service experience phase often leads to a significant time delay between purchase and delivery [23]. Consumers may tend to view waiting and adjustment costs as inherent to online pre-sales. Additionally, they may expect a certain level of uncertainty or adjustment [11], which could make them more accepting of these costs than initially anticipated. As a result, this may reduce the expected moderating effects of product type on waiting and adjustment costs. Secondly, we believe that consumer tolerance for waiting or adjustment costs may vary based on individual characteristics, such as prior purchase experience or brand trust [20]. For instance, consumers with high brand trust may have their perception of waiting and adjustment costs buffered, which could explain why the results were not significant.

## 7. Implications and Limitations

### 7.1. Theoretical Implications

Firstly, this article primarily investigates the influencing factors of consumers’ continuous participation intention during an online pre-sale. Most previous studies on continuous participation in online shopping have been based on the context of spot sales [32,45,75], without considering the adaptability of the influencing factors of consumers’ continuous participation intention under the new model of online pre-sales. The online pre-sale is apparently different from other online sales in terms of price discount presentation and product delivery time [11,23]. These differences will inevitably impact consumers’ perceived benefits and perceived costs, and subsequently their continuous participation intention. Moreover, current academic studies on online pre-sales mostly adopt the perspective of the merchant, conducting qualitative research on pricing issues, supply chain decision-making, and optimal pre-sale strategy formulation in online pre-sales [7,8,22]. There is relatively less empirical data analysis research on consumers’ participation in pre-sale activities. Therefore, this study explores the influencing factors of consumers’ continuous participation intention during an online pre-sale by combining the characteristics of online pre-sale and adopting the perspective of consumers. Our study further expands the research on consumers’ continuous participation intention in online pre-sale mode and investigates the relationship between consumers’ psychological perceptions and their continuous participation intention, filling the research gap in empirical analysis related to online pre-sale.

Secondly, this study applies social exchange theory to the study of consumers’ continuous participation intention. Based on the social exchange theory, consumers’ perceived value is divided into perceived costs and perceived benefits. Moreover, different dimensions of the perceived value are further explored for their impact on consumers’ satisfaction and willingness to continue to participate. Previous studies have explored the relationship between perceived value and the willingness to continue participating mainly from the perspectives of uses and gratifications theory, measures-end chain theory, prospect theory, SOR theory, and cost-benefit theory [33,38,39]. However, under the social exchange theory, we believe that consumers’ continuous participation in online pre-sale activities is a kind of exchange behavior [17]. When consumers perceive benefits in online pre-sale activities, they reciprocate in the form of continuous participation. Thus, this study investigates the perceived costs and benefits of consumers’ participation in online pre-sale activities, combines them with the characteristics of online pre-sale activities, and divides perceived benefits and perceived costs into three dimensions, respectively. We explore their effects on consumers’ satisfaction and willingness to continue participating and broaden the application of social exchange theory in social commerce.

Finally, this study demonstrates that product type plays a moderating function in the dimensions of perceived benefits, perceived costs, and consumer satisfaction in the online pre-sale, broadening the boundaries between perceived benefits, perceived costs, and consumer satisfaction. Previous studies have considered the impact of individual consumer differences [117], but have ignored the differences in consumers’ perceived value when purchasing different types of products. This paper effectively explains this research divergence by introducing product types. This is a crucial theoretical construct to better explain the interaction mechanism between perceived value and customer satisfaction. It also echoes the conclusion made by [41,118] for product type to have a major impact on consumer behaviors.

### 7.2. Practical Implications

The practical significance of this study is as follows and some suggestions based on our research findings are also listed:

Firstly, businesses can enhance consumers’ perceived benefits to strengthen their willingness to continuously participate in online pre-sale activities in the following ways: (1) strengthen consumers’ utility benefits: merchants should ensure the quality of online pre-sale products while providing price discounts, improving consumers’ experience in participating in pre-sale activities. (2) Focus on consumers’ hedonic benefits: the pleasure and relaxation that consumers perceive in online pre-sale activities will have a positive impact on their continuous participation intention. Merchants can enhance consumers’ hedonic benefits by improving the aesthetics of the online pre-sale activity interface, constructing game zones, and other methods. (3) Pay attention to consumers’ social benefits: under the trend of e-commerce, consumers are not only satisfied with utility and hedonic benefits but also begin to pursue social benefits. Merchants can design interactive modules to promote communication between consumers and between consumers and merchants, which enhances consumers’ sense of identity.

Secondly, businesses should try to reduce consumers’ perceived costs in the following ways: (1) optimize the shopping interface settings: businesses should ensure the clarity and completeness of the information released to help consumers quickly target the product information they need, thus reducing their perceived search costs. (2) Set reasonable pre-sale and delivery times: businesses should reduce the waiting cost of consumers in the pre-sale situation to avoid the adverse consequences of consumers waiting for a long time, resulting in lower sales or loss of customers. (3) Consider consumers’ perception of adjustment costs: real and detailed descriptions of online pre-sale products can reduce information asymmetry and consumer uncertainty about product valuation and reduce the occurrence of after-sale adjustment events such as changes in user orders. Therefore, businesses should improve the consistency of product descriptions and avoid false advertising.

Finally, according to the findings of this paper, businesses should invest more resources or costs to promote experience-based products than search-based products. (1) Invest in marketing campaigns: Businesses might invest more money and personnel in marketing campaigns, like Taobao Express, to promote experience-based products. (2) Conduct market research: Businesses should perform market research on their target audience before pushing different types of online pre-sale products to clarify the perceived benefits and cost avoidance that consumers expect to gain by continuing to acquire pre-sale products.

By focusing on these strategies, businesses can better cater to consumers’ expectations and preferences, ultimately leading to increased consumer satisfaction and a higher likelihood of continuous participation in online pre-sale activities.

### 7.3. Limitations and Future Research Directions

This study does not explicitly differentiate between consumers’ payment methods. However, in the online pre-sale, consumers’ payment methods may also affect their preferences and behaviors [119]. In follow-up research, we will distinguish between deposits and full prepayments and investigate the impact of various prepayment methods on consumers’ willingness to continuously participate in online pre-sale activities. Secondly, this study obtained data through questionnaire surveys. Although the samples collected in this study basically cover the whole area of China and can meet the needs of empirical analysis in the article, the generalizability of the research results is limited due to the large proportion of respondents aged 19–25. In the future, we can consider cooperating with e-commerce platforms to access their user databases and directly collect user transaction data during the entire pre-sale period, thereby enhancing the credibility of the research findings. Finally, future research could more fully consider the impact of individual differences in consumer characteristics and explore the distinctions and connections between online pre-sales and other forms of online sales. This would help enrich the current research framework.

## Figures and Tables

**Table 1:** The profile of the respondents.

Demographic Characteristics	Type	Frequency (*n* = 527)	%
Gender	Male	220	41.7%
	Female	307	58.3%
Age	<18	3	0.6%
	19~25	430	81.6%
	26~35	88	16.7%
	>36	6	1.1%
Education	High school and below	5	0.9%
	College	45	8.5%
	Undergraduate	370	70.2%
	Master’s degree and above	107	20.3%
Online shopping experience (year)	<1	4	0.8%
	1–3	92	17.5%
	3–5	205	38.9%
	>5	226	42.9%
Job	Students	431	81.8%
	Party and government organizations and public institution staff	21	4.0%
	Enterprise, company staff	56	10.6%
	Farmers	3	0.6%
	Freelance	15	2.8%
	Other	1	0.2%

**Table 2:** Reliability and validity analysis.

	Item	CITC	Cronbach’s Alpha if Item Deleted	Standardized Factor Load Values	Cronbach’s α	AVE	CR	UB	HB	SB	SC	WC	AC	CS	CPI
UB	UB 1	0.852	0.911	0.891	0.933	0.823	0.933	0.907							
	UB 2	0.871	0.895	0.920											
	UB 3	0.863	0.901	0.911											
HB	HB 1	0.844	0.920	0.882	0.937	0.789	0.937	0.074	0.888						
	HB 2	0.854	0.917	0.889											
	HB 3	0.850	0.918	0.887											
	HB 4	0.857	0.916	0.895											
SB	SB 1	0.818	0.905	0.858	0.925	0.755	0.925	0.147	0.127	0.869					
	SB 2	0.818	0.905	0.861											
	SB 3	0.827	0.902	0.870											
	SB 4	0.839	0.898	0.886											
SC	SC 1	0.838	0.913	0.891	0.93	0.816	0.930	0.185	0.057	0.169	0.903				
	SC 2	0.863	0.894	0.920											
	SC 3	0.868	0.889	0.911											
WC	WC 1	0.837	0.899	0.884	0.925	0.805	0.925	0.135	−0.18	0.049	0.056	0.897			
	WC 2	0.849	0.889	0.901											
	WC 3	0.854	0.885	0.906											
AC	AC 1	0.818	0.901	0.864	0.92	0.795	0.921	0.085	0.182	0.163	0	0.169	0.892		
	AC 2	0.857	0.869	0.917											
	AC 3	0.840	0.884	0.893											
CS	CS 1	0.851	0.927	0.886	0.938	0.837	0.939	0.224	0.279	0.282	0.228	0.256	−0.245	0.915	
	CS 2	0.885	0.899	0.932											
	CS 3	0.881	0.904	0.926											
CPI	CPI 1	0.654	0.688	0.821	0.788	0.577	0.800	0.393	0.339	0.377	0.395	0.376	−0.339	0.532	0.760
	CPI 2	0.690	0.656	0.829											
	CPI 3	0.562	0.802	0.607											

**Table 3:** CFA model fitness test.

Indicators	Reference Standards	Measurement Results
CMIN/DF	1–3 is excellent, 3–5 is good	1.193
RMSEA	<0.05 is excellent, <0.08 is good	0.019
IFI	>0.9 is excellent, >0.8 is good	0.995
TLI	>0.9 is excellent, >0.8 is good	0.994
CFI	>0.9 is excellent, >0.8 is good	0.995

**Table 4:** Structural model path relationship analysis and hypothesis testing.

Path	Estimate	S.E.	C.R.	*p*	Hypothetical Results
UB → CS	0.143	0.047	3.057	0.002	H1a Supported
HB → CS	0.227	0.05	4.503	***	H1b Supported
SB → CS	0.249	0.055	4.497	***	H1c Supported
SC → CS	−0.176	0.046	−3.815	***	H2a Supported
WC → CS	−0.195	0.048	−4.067	***	H2b Supported
AC → CS	−0.18	0.05	−3.583	***	H2c Supported
UB → CPI	0.111	0.019	5.774	***	H3a Supported
HB → CPI	0.085	0.021	4.125	***	H3b Supported
SB → CPI	0.109	0.023	4.778	***	H3c Supported
SC → CPI	−0.126	0.019	−6.606	***	H4a Supported
WC → CPI	−0.109	0.02	−5.493	***	H4b Supported
AC → CPI	−0.094	0.02	−4.603	***	H4c Supported
CS → CPI	0.106	0.019	5.455	***	H5 Supported

Note: *** *p*< 0.001.

**Table 5:** Tests for the moderating effects of product type.

	Search-Based				Experience-Based				Difference Examination	
	Unstandardized Coefficients		Standardized Coefficients	*t*	Unstandardized Coefficients		Standardized Coefficients	*t*	|Z|	Hypothetical Results
Path	B	Std. Error	Beta		B	Std. Error	Beta			
UB → CS	0.009	0.068	0.007	0.125	0.205	0.049	0.188	4.174	2.338 *	H6a Supported
HB → CS	0.002	0.078	0.002	0.031	0.323	0.055	0.26	5.884	3.363 ***	H6b Supported
SB → CS	0.069	0.086	0.048	0.803	0.443	0.055	0.367	8.078	3.664 ***	H6c Supported
SC → CS	−0.029	0.066	−0.026	−0.44	−0.288	0.051	−0.247	−5.609	3.105 **	H7a Supported
WC → CS	−0.11	0.07	−0.094	−1.576	−0.053	0.056	−0.045	−0.939	0.636	H7b Unsupported
AC → CS	−0.046	0.073	−0.038	−0.632	−0.189	0.053	−0.166	−3.552	1.585	H7c Unsupported

Note: *** *p*< 0.001; **p < 0.01; * *p* < 0.05.

**Table 6:** Result (*n* = 527).

	Model 1		Model 2		Model 3	
	B	*t*	B	*t*	B	*t*
constant	4.250 ***	9.354	4.255 ***	10.499	4.272 ***	10.985
Gender	−0.106	−0.865	−0.046	−0.417	−0.016	−0.149
age	−0.046	−0.341	−0.06	−0.491	−0.039	−0.338
edu	−0.069	−0.72	−0.105	−1.225	−0.146	−1.767
exper	0.043	0.629	0.046	0.761	0.049	0.854
job	−0.003	−0.056	−0.007	−0.13	−0.007	−0.143
UB			0.136 **	3.023	0.001	0.024
SB			0.241 ***	4.447	0.077	1.034
HB			0.225 ***	4.432	0.003	0.046
SC			−0.158 ***	−3.509	−0.022	−0.387
WC			−0.183 ***	−3.912	−0.103	−1.707
AC			−0.160 ***	−3.358	−0.043	−0.698
TYPE			0.053	0.615	0.058	0.699
UB*TYPE					0.210 *	2.344
SB*TYPE					0.252 *	2.387
HB*TYPE					0.439 ***	4.378
SC*TYPE					−0.269 **	−2.99
WC*TYPE					0.045	0.461
AC*TYPE					−0.146	−1.532
R 2	0.003		0.222		0.297	
F	F (5521) = 0.292, *p* = 0.917		F (12,514) = 12.213, *p* = 0.000		F (18,508) = 11.900, *p* = 0.000	
∆R 2	0.003		0.219		0.075	
∆F	F (5521) = 0.292, *p* = 0.917		F (7514) = 20.672, *p* = 0.000		F (6508) = 8.995, *p* = 0.000	

Dependent variable: CS. * *p* < 0.05; ** *p* < 0.01; *** *p* < 0.001.

## Data Availability

The raw data supporting the conclusions of this article will be made available by the authors upon request.

## Appendix A

**Table A1:** Variable Measurement Items and Sources.

Variable	Item	Source
Utility benefit (UB)	I feel that shopping during an online pre-sale is good value for money	[62,102]
	I can avoid the risk of out-of-stock items by shopping via online pre-sales	
	I have a more personalized choice of products in the online pre-sale	
Hedonic benefit (HB)	I find online pre-sales on e-commerce platforms very interesting	[62,102]
	I enjoy the online pre-sale shopping model	
	Participating in online pre-sales makes me feel good	
	I feel engaged when browsing the pre-sale goods	
Social benefit (SB)	I can keep up with fashion trends by shopping through online pre-sales	[62,120]
	I feel like I have something to talk about with other users when I participate in online pre-sales	
	I communicate and share my experiences with others when I participate in online pre-sales	
	I feel a sense of achievement when I share my experiences of online pre-sales with other users	
Search cost (SC)	It takes me a long time to search for the details of pre-sale goods	[45]
	In the online pre-sale, sellers have a limited time frame for the pre-sale	
	I have to put a lot of effort into sifting through valuable information for online pre-sales	
Waiting cost (WC)	I have to wait a while for the final payment after paying a deposit for some pre-sale orders	[44,46]
	I have to wait a long time for my order to be shipped when I shop in online pre-sale mode	
	When I shop online, it takes longer than I expected for my order to be delivered	
Adjustment cost (AC)	Changing my pre-sale order will take more time and effort than other online shopping models	[45]
	It is more trouble to solve problems with merchants not delivering on time in the online pre-sale	
	It will take me more time and effort to return a pre-sale product if it does not arrive as expected	
Consumer satisfaction (CS)	I think it is a wise choice to shop through an online pre-sale	[102]
	Overall, the products and services provided during an online pre-sale met my expectations	
	I am generally satisfied with the online pre-sale	
Consumer continuous participation intention (CPI)	I would like to continue to participate in online pre-sales and purchase products again	[75]
	I would recommend the online pre-sale to others	
	I would encourage my friends and family to take part in an online pre-sale	
